# Converge or Collide? Making Sense of a Plethora of Open Data Standards in Health Care

**DOI:** 10.2196/55779

**Published:** 2024-04-09

**Authors:** Guy Tsafnat, Rachel Dunscombe, Davera Gabriel, Grahame Grieve, Christian Reich

**Affiliations:** 1 Evidentli Pty Ltd Surry Hills Australia; 2 Centre for Health Informatics Australian Institute of Health Innovation Macquarie Univeristy Macquarie Park Australia; 3 OHDSI OMOP + FHIR Working Group; 4 openEHR International St. Helens United Kingdom; 5 Imperial College London London United Kingdom; 6 School of Medicine Johns Hopkins University Baltimore, MD United States; 7 Health Level 7 International Ann Arbor, MI United States; 8 Health Intersections Pty Ltd Melbourne Australia; 9 Odysseus Data Services Cambridge, MA United States

**Keywords:** interoperability, clinical data, open data standards, health care, digital health, health care data

## Abstract

Practitioners of digital health are familiar with disjointed data environments that often inhibit effective communication among different elements of the ecosystem. This fragmentation leads in turn to issues such as inconsistencies in services versus payments, wastage, and notably, care delivered being less than best-practice. Despite the long-standing recognition of interoperable data as a potential solution, efforts in achieving interoperability have been disjointed and inconsistent, resulting in numerous incompatible standards, despite the widespread agreement that fewer standards would enhance interoperability. This paper introduces a framework for understanding health care data needs, discussing the challenges and opportunities of open data standards in the field. It emphasizes the necessity of acknowledging diverse data standards, each catering to specific viewpoints and needs, while proposing a categorization of health care data into three domains, each with its distinct characteristics and challenges, along with outlining overarching design requirements applicable to all domains and specific requirements unique to each domain.

## Introduction

The digital health sector presents unique and diverse data challenges that manifest as a fractured ecosystem and often fails to deliver effective collaboration among interconnected entities [1]. The consequences include inconsistencies between services rendered and payments, waste, and—most importantly—suboptimal care. Interoperable data has been hailed as a near-future solution to many of these challenges for decades. Paradoxically, interoperability efforts have been fractured and inconsistent, resulting in many incompatible interoperability standards, despite widespread acknowledgment that fewer standards would provide better interoperability [2,3]. This paper presents a typology of healthcare data requirements and describes the challenges and opportunities of open data standards in health care.

Recognizing that different data standards represent different points of view and respond to different needs, and that no single standard would necessarily be able to meet all the requirements of all health care systems, we distinguish three domains of health care data with their unique characteristics and challenges, and outline high-level design requirements. We distinguish between requirements that are common across all domains and those that are specific to each domain.

## Three Domains of Health Care Data

### Clinical Care and Administration

Health care data are generated as documentation of clinical service delivery for various purposes, including continuity of care and billing. A repository of clinical records containing the complete patient history of every patient is required to deliver continuous and consistent care efficiently and effectively. As clinical care sometimes lacks a strong evidence base from which best practices can be gleaned, care documentation should be able to address a broad range of data types, including “one off” events reflecting unforeseen patient circumstances [4].

The main focus of standards for electronic medical record interoperability is to protect users from vendor lock-in, in which the cost of changing to a better system outweighs the benefits expected from the switch. An electronic health care record system that uses an open standard can replace another that uses the same standard far more easily and with less disruption to clinical practice than two incompatible systems where data transformation from one system to the next represents the lion’s share of the cost.

### Data Exchange

Records of care provided are used in communication between different actors. For example, continuity of care depends on multiple health care providers sharing information about the patient, and effective billing requires sharing information between providers and payers. It is often critical that information is passed between systems immediately to inform urgent decisions. Standards for the transmission of health care data should be able to be arranged in small quantities that include all and only the relevant information.

The main focus of data exchange standards is fidelity to the operational health care processes. For example, one focus is on capturing and faithfully representing the terminology used in the local processes, whatever that is, as opposed to restricting use to a common terminology.

### Longitudinal Analysis

The main focus of longitudinal analysis are patterns, trends, and their predictors. A practical consideration is that such patterns tend to be subtle, and hence, data from multiple systems and organizations are required to be aggregated or compared to elucidate reliable phenomena. As an empirical discipline, medicine has incrementally improved using inferences made from big data. Moreover, medical evidence should produce predictable outcomes across multiple settings and for all patients.

Analytical methods use generalizations such as grouping patients with similar characteristics like age. “One off” events and subtle differences in clinical practice are inconsequential or even harmful to population statistics. For example, as age is often measured in years and stratified into a larger age bracket, the exact birth date of each patient can be substituted by an approximate date without changing the results of the analysis. Another common reason to lose fidelity is patient privacy and prevention of unauthorized reidentification of individuals from analytic data sets.

## Practicality, Feasibility, and Community

### Technical Trade-Offs

The technical suitability of a standard to the problem it addresses is important, but digital health and data interoperability are applied disciplines. Practical and socio-technical considerations, such as available skill sets, are as important as, if not more important than, technical superiority. While no standard is perfect, there are major differences in what standards can do “out of the box” versus how much customization is required before they can be put to use. To provide data interoperability in practice, standards have to find the right balance between customizability and rigidity. The United States Core Data for Interoperability is a prime example of such balance [5]. These core data element definitions are regulated for use in the United States and can be thought of as a midpoint between customizations needed to be made at every site and rigidity with regard to specific data elements.

### Community

Data standards are only as useful as their adoption by collaborating organizations, so standards that are in widespread use in the community are more likely to be beneficial to organizations seeking to join collaboration networks that use these standards. Engagement from communities that not only use a standard but also contribute and improve it over time, differentiates legacy standards from more sustainable ones. An actively engaged community will, over time, evolve even an infantile standard into a superior one. An engaged and vibrant community is therefore a major advantage for the longevity of the data standards it uses [6]. Open standards have a clear advantage over proprietary ones, as they are available for contribution from a larger audience and are tested in more diverse environments. Conversely, it is usually easier to govern the development of proprietary standards, which can lead to shorter improvement cycles [7].

## Terminologies

A common aspect of all data standards, regardless of application, is the use of common terminologies. Many standards rely on terminologies that are developed independently. Terminologies can be flat or ontological, specific to one or a handful of domains or more general, proprietary or open. Depending on the intended use of the standard and available resources, the inclusion policy for terminologies in a standard may differ. Standards designed for continuity of care may allow the use of external terminologies if they are available to all parties providing care, whereas standards used for data mining may restrict their use to only vetted terminologies. As is the case of data standards, terminologies benefit from active communities to keep them up to date. As evident in differing design criteria, a truly interoperable, agile health care system that provides the best possible clinical care implements multiple standards. For example, standard records from a repository of complete patient histories may be transformed to a standard for transmission and transformed again when entered into a data warehouse for analysis. Systematic harmonization between standards would reduce or eliminate information loss in the transformation processes [8].

## Emerging Standards

### Paradox of Interoperability

A paradox of health care interoperability is the existence of a large number of standards with significant overlap among them. Each may be more or less equally suited for its intended use, so selecting a standard for implementation can be difficult. Harmonization from all standards to all others is impractical. The interoperability paradox is that more standards lead to less interoperability. We therefore propose that the health informatics community converge on just three standards. We have chosen standards that are open, are backed by active communities, and have been tried and tested in their respective areas.

### Clinical Care and Administration

openEHR [9] provides a common data structure for health IT systems, including but not limited to, electronic medical records and patient administration systems. The standard uses a centralized database with a tree structure for efficient retrieval of a complete patient record. The schema of the database is patient-centric, clinical, and extensible with reusable archetypes and templates. openEHR is increasingly preferred over proprietary formats, as multiple vendors that offer commercial systems based on openEHR means that a very expensive process for switching vendors can be greatly simplified.

### Data Exchange

Health Level 7’s (HL7) Fast Healthcare Interoperability Resources (FHIR) [10] is an open standard particularly suited for data transfer. At the center of the FHIR specification is a representational state transfer (RESTful) application programming interface (API) that provides real-world health care services such as “record patient details,” “prescribe a medication,” “suggest the correct treatment for a patient,” and “make an appointment with your doctor.” Its resource structure only contains information relevant to a particular API call so it can be transmitted efficiently. Specific minimum data sets of FHIR (called cores) are required in the United States, with other countries currently developing additional core requirements. Using FHIR allows different entities in that country to streamline continuity of care by exchanging information between systems and organizations.

### Longitudinal Analysis

Observational Health Data Sciences and Informatics’ (OHDSI) Observational Medical Outcomes Partnership Common Data Model (OMOP) [11] is an open standard designed for longitudinal interrogation of clinical records. Its use of a patient-centric relational model makes it suited for complex queries across many patients’ records to discover patterns and trends using statistical and machine learning techniques.

As the requirements for each data standard mean that one standard cannot replace the others, harmonization projects are led by community volunteers to provide standard transformations from one standard to another. For example, the collaboration between OHDSI and HL7 manifests as a common working group with weekly meetings to produce an implementation guide for algorithms that convert data from FHIR to OMOP and from OMOP to FHIR. Similar projects for harmonizing openEHR with OMOP and FHIR with openEHR provide a standard architecture for effective data interoperability and reuse that will create a virtuous cycle of growing community participation, improving the standards and simplifying harmonization. Such volunteer groups are highly beneficial, not only to the standards they are dedicated to but also to health care systems globally.

### Which Standard to Use?

**Figure 1:**
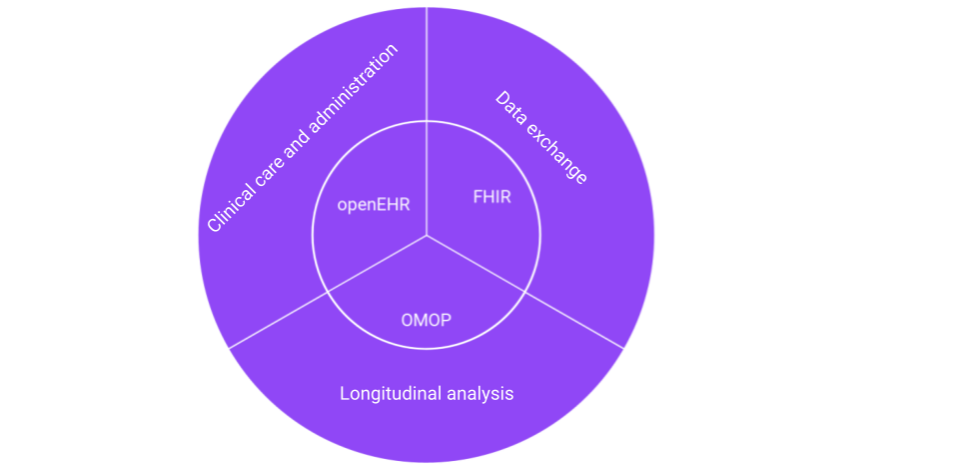
Alignment between domain (outer circle) and open standard (inner circle). FHIR: Fast Healthcare Interoperability Resources; OMOP: Outcomes Partnership Common Data Model.

Most users will easily choose whether their needs’ focus is operational care delivery or analytics and identify which systems are involved. Figure 1 provides a high-level key, making it easy to choose between openEHR, OMOP, or FHIR and invest in extract, transform, load processes to change data representations from one to another. An interoperable health system would use openEHR to collect data, FHIR to transmit data between systems and organizations, and OMOP to find insights in the data.

It is possible for transformations from one standard to another to be “lossy.” For example:

- Fidelity may be lost when converting from a specific term (eg, “fracture of the fourth metatarsal on the left foot”) to a more general one (eg, “fracture of the fourth metatarsal”).
- Information can be lost when information captured in one standard (eg, the time drug administration was witnessed in FHIR) has no place in another standard (eg, OMOP).
- Relationships may be lost when information has to be split (eg, a diagnosis and subsequent treatment).
- Accuracy may be lost when converting to a standard that requires information not found in the other (eg, the time a drug was stopped is required for every exposure in OMOP and may need to be estimated whenever it is not present in the data set).

Consideration should be given to minimizing such losses. These approaches can use tacit or implicit knowledge about the data set, external knowledge, or metadata [12].

## Conclusion

Open standards, backed by engaged communities, hold an advantage over proprietary ones. They enable broader contributions and testing, fostering continual improvement. However, achieving true interoperability requires harmonization among standards and a pathway for migration from other standards and proprietary models. Three open standards are particularly suited to their respective health care domains: openEHR for clinical care and administration, FHIR for data exchange, and OMOP for longitudinal analysis. The most important aspect of every standard is its community, and all three have active and growing communities that continue to use and improve these standards.

## Abbreviations

API: application programming interface
FHIR: Fast Healthcare Interoperability Resources
HL7: Health Level 7
OHDSI: Observational Health Data Sciences and Informatics
OMOP: Outcomes Partnership Common Data Model
RESTful: representational state transfer
